# Transplant Outcomes After Exposure of Deceased Kidney Donors to Contrast Medium

**DOI:** 10.1097/TP.0000000000004745

**Published:** 2023-09-06

**Authors:** Kinita A. Chotkan, Luuk B. Hilbrands, Hein Putter, Cynthia Konjin, Brigitte Schaefer, Ludo F.M. Beenen, Robert A. Pol, Andries E. Braat

**Affiliations:** 1 Department of Surgery, Leiden University Medical Center, Leiden, the Netherlands.; 2 Department of Organ and Tissue Donation, Dutch Transplantation Foundation, Leiden, the Netherlands.; 3 Department of Nephrology, Radboud University Medical Center, Nijmegen, the Netherlands.; 4 Department of Biomedical Data Sciences, Leiden University Medical Center, Leiden, the Netherlands.; 5 Department of Radiology and Nuclear Medicine, Amsterdam University Medical Center, Amsterdam, the Netherlands.; 6 Department of Surgery, Division of Transplantation, University Medical Center Groningen, Groningen, the Netherlands.

## Abstract

**Background.:**

The administration of contrast medium is associated with acute kidney injury; however, the effect of exposure of a deceased organ donor to contrast medium on kidney transplant outcomes has been poorly studied.

**Methods.:**

A retrospective analysis of all deceased kidney donors between 2011 and 2021 and their corresponding recipients in the Netherlands was conducted. Multivariable analyses were performed to assess the associations between contrast medium exposure and delayed graft function (DGF)/graft survival. Linear mixed models were used to assess the differences in mean estimated glomerular filtration rate values in recipients 1 to 6 y after transplantation.

**Results.:**

In total, 2177 donors and 3638 corresponding kidney graft recipients were included. Twenty-four percent of the donors (n = 520) were exposed to contrast medium, corresponding to 23% of recipients (n = 832). DGF was observed in 36% (n = 1321) and primary nonfunction in 3% (n = 122) of recipients. DGF rates for donation after brain death (DBD) and donation after circulatory death (DCD) donors showed no significant effect of contrast medium exposure (*P* = 0.15 and *P* = 0.60 for DBD and DCD donors, respectively). In multivariable analyses, contrast medium administration was not significantly associated with a higher DGF risk (odds ratio 1.06; 95% confidence interval, 0.86-1.36; *P* = 0.63) nor was a significant predictor for death-censored graft failure (hazard ratio 1.01; 95% confidence interval, 0.77-1.33; *P* = 0.93). Linear mixed models showed no difference in mean estimated glomerular filtration rate values in recipients 1 to 6 y posttransplantation (*P* = 0.78).

**Conclusions.:**

This study indicates that contrast medium administration in DBD and DCD donors has no negative effect on early and long-term kidney graft function.

## Graphical abstract

**Figure:**
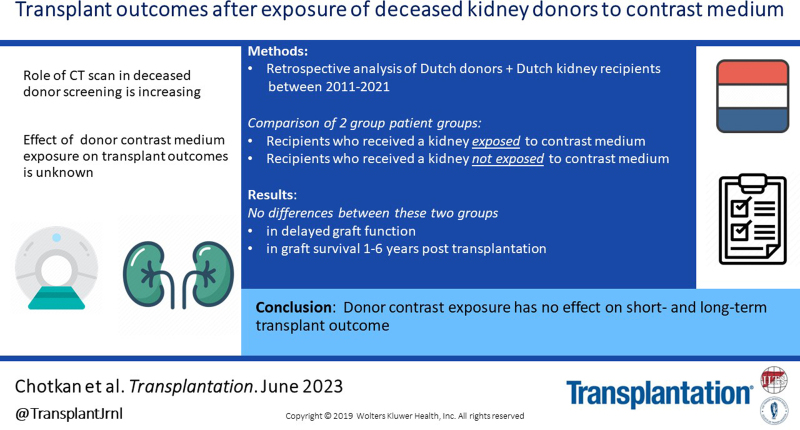

## INTRODUCTION

Computed tomography (CT) has gained importance as a diagnostic imaging method in the deceased donor screening process.^1-3^ Detailed imaging helps to evaluate vascular anatomy, may detect malignancies, allows assessment of organ quality, and improves size matching in liver and lung transplantation.^1,2,4-7^ In the Eurotransplant region, chest x-ray and abdominal ultrasound are the minimal required diagnostic tools, but a recent review of all deceased donors in the Netherlands showed that a CT scan is performed in 18% of the cases during donor workup or at admission.^3,8^ Because of the many advantages of performing CT scans, it is not unrealistic to assume that CT scans will become standard protocol in the evaluation of potential deceased organ donors in the future, as is already the case in several countries.^1,5,9-11^ The administration of intravenous (IV)-contrast medium is often necessary to adequately visualize the (vascular) anatomy or pathological conditions on a CT scan. However, the administration of IV-contrast medium has been associated with contrast-induced acute kidney injury (CI-AKI). CI-AKI is the occurrence of kidney injury within 72 h after the administration of iodinated contrast media, measured as an increase in creatinine level by >25% or 0.5 mg/dL.^12,13^ The mechanism of CI-AKI is not completely understood but is most likely a combination of the cytotoxic effects of the contrast medium on tubular cells and vasoconstriction leading to acute tubular necrosis.^14,15^ Although a meta-analysis of 107 335 patients concluded that the risk of CI-AKI after IV-contrast medium administration might be lower than previously reported, a consensus on the actual risk has not yet been reached.^16^

For the functional and anatomical assessment of potential heart donors, coronary angiography with intra-arterial (IA) contrast medium or coronary CT angiography with IV-contrast medium administration is necessary in some cases. Coronary angiography usually requires a higher contrast medium volume compared with coronary CT angiography.^17^ Literature comparing the risk of developing CI-AKI after IA-contrast medium and IV-contrast medium administration shows varying results. A prospective randomized controlled trial investigating the impact of IA- and IV-contrast medium administration on CI-AKI development showed that AKI was more common after IA-contrast medium administration (13.2% versus 5.6%, *P* = 0.02).^18^ However, McDonald et al^17^ found no significant difference in the AKI rate after IA- or IV-contrast medium administration (11% versus 9.9%, respectively, *P* = 0.12).

The effects of IA- and IV-contrast medium administration to donors on graft function in kidney transplant recipients have been poorly investigated. Studies have been subjected to selection bias, including a relatively young donor population and donation after brain death (DBD) donors in most studies.^19-22^ This does not reflect the development of the donor pool in the Eurotransplant region, in which the donor age and the number of donations after circulatory death (DCDs) donors are increasing.^23,24^ DCD kidneys are at a higher risk of ischemia–reperfusion injury and delayed graft function (DGF) because of the exposure to additional warm ischemia caused by the lack of blood perfusion of organs during the agonal phase and after circulatory arrest, compared with DBD kidneys (which are not exposed to warm ischemia).^25,26^ This could make DCD kidneys more susceptible to CI-AKI; however, this has not yet been sufficiently proven.

Gaining more knowledge about the effect of exposing DBD and DCD donors to contrast medium on transplant outcomes is becoming increasingly important, given the increase in contrast medium–dependent diagnostics used during the donor screening process. Therefore, this study aimed to investigate the effects of IA- and IV-contrast medium administration during deceased donor assessment on short- and long-term kidney transplant outcomes.

## MATERIAL AND METHODS

### Study Design and Population

This study was a retrospective review of all Dutch deceased kidney donors reported to Eurotransplant between January 2011 and June 2021 and all corresponding Dutch kidney graft recipients. Exclusion criteria were DCD donors categories I (dead upon arrival at the hospital) and II (unsuccessfully resuscitated donors), kidneys that were transplanted abroad, resulting in incomplete follow-up data, donors of whom both kidneys were discarded, or when information was missing on exposure to contrast medium.^27^

Since 2016, kidneys from Dutch donors that have been transplanted in Dutch transplant centers have been subjected to hypothermic machine perfusion, the type of perfusion machine (LifePort Kidney Transporter-Organ Recovery Systems or Kidney Assist Transport-Organ Assist), depending on the type of donor. One of both kidneys of DBD donors and one of both kidneys of DCD donors aged <50 y were placed on the LifePort Kidney Transporter (not oxygenated hypothermic machine perfusion). The paired kidney was placed on the Kidney Assist Transporter, as well as both kidneys from DCD donors aged ≥50 y. In DCD donors aged >50 y, oxygenated hypothermic machine perfusion was applied using the Kidney Assist Transporter.^28^

### Data Source

Baseline characteristics of donors were obtained from the Eurotransplant Network Information System and Organ Procurement Information Database of the Dutch Transplantation Foundation (Nederlandse Transplantatie Stichting), which are mandatory registries. Follow-up data of kidney graft recipients were obtained from the Netherlands Organ Transplant Registry (NOTR). For each kidney transplant, donor records were reviewed for exposure to either IV- or IA-contrast medium during hospital admission.

In the Eurotransplant database, no information regarding the indication for the CT scan was registered. The indication for coronary angiography in the study cohort was solely to determine the suitability of the heart for donation. Performing coronary angiography is not a standard procedure but can be requested by the transplant center, for example, in patients with multiple comorbidities (ie, a history of hypertension or a history of smoking).

The study protocol was approved by the review board of the NOTR (registration no. 51588) and conducted in accordance with the World Medical Association Declaration of Helsinki and the Declaration of Istanbul.

### Definitions and Study Endpoints

The primary outcome measures were the incidence of primary nonfunction (PNF) and the incidence and duration of DGF. Secondary outcome measures included death-censored graft survival and yearly estimated glomerular filtration rate (eGFR) up to 6 y after kidney transplantation.

PNF was defined as a nonfunctioning kidney allograft 3 mo posttransplantation. DGF was defined as the requirement for dialysis within the first 7 d after transplantation, excluding patients with PNF. The first warm ischemic time was defined as the time from asystole in the DCD donor until the start of cold perfusion. Cold ischemic time was defined as the time from the start of cold perfusion until removal from cold storage or cold machine perfusion in the transplant center. The second warm ischemic time was defined as the time from kidney removal from static cold storage or hypothermic machine perfusion until reperfusion in the recipient.^8^

The Modification of Diet in Renal Disease equation (without ethnicity) was used to calculate the eGFR in mL/min/1.73 m^2^.^29^

### Kidney Donor Risk Index

The Kidney Donor Risk Index (KDRI), donor only, was calculated using a standardized formula that includes age (years), height (cm), weight (kg), history of hypertension (yes/no), history of diabetes (yes/no), cause of death (cerebrovascular accident, yes/no), serum creatinine (mg/dL), DCD status, and hepatitis C virus status.^30,31^ Ethnicity was not available in the database; therefore, all donors were categorized as Caucasian by default.

### Statistical Analysis

Continuous data are presented as mean ± SD or median with interquartile range. Unless stated otherwise, categorical data are presented as absolute numbers and percentages (%). The Shapiro-Wilk test was used to assess whether the continuous variables were normally distributed. Continuous variables with a normal distribution were assessed using parametric tests, and nonparametric tests were used for variables showing a skewed distribution. Differences between categorical data were assessed using the chi-square tests. A *P* value of <0.05 was considered statistically significant.

Univariable and multivariable stepwise binary logistic regression analyses were used to evaluate the associations between donor, recipient, and procedural characteristics with DGF. Kaplan-Meier survival curves were used to calculate death-censored graft survival, and the log-rank test was used to assess the difference in death-censored graft survival 5 y posttransplantation between no-contrast medium exposure (contrast medium^–^) and contrast medium exposure (contrast medium^+^). Univariable and stepwise Cox regression analyses were performed to assess the associations between donor, recipient, and procedural characteristics and death-censored kidney graft survival. The results are presented as odds ratios (ORs) or hazard ratios (HRs) with corresponding confidence intervals (CIs) and *P* values.

Linear mixed models were used to assess the mean change in kidney function (expressed as eGFR) during the first 6 y posttransplantation. To assess the longitudinal effect of contrast medium^–^ versus contrast medium^+^ on eGFR, we defined contrast medium exposure, posttransplant time in years, and the interaction between contrast medium exposure and posttransplant time as fixed effects.

Separate analyses were performed for donors with an eGFR <60 mL/min/1.73 m^2^ and donors who underwent coronary angiography. To compare donors who underwent coronary angiography with those who did not undergo coronary angiography, only DBD donors with exclusion of “kidney only donors” were analyzed.

To investigate whether potential kidney donors who were not accepted for transplantation and were exposed to contrast medium developed acute kidney injury (and were therefore rejected for transplantation), the reasons for not accepting a potential kidney donor were reviewed. This information was only registered since 2014. For the statistical analyses, IBM SPSS Statistics for Windows was used (IBM Corp. Released 2016, version 24.0. Armonk, NY).

## RESULTS

### Donor Characteristics

**FIGURE 1.**
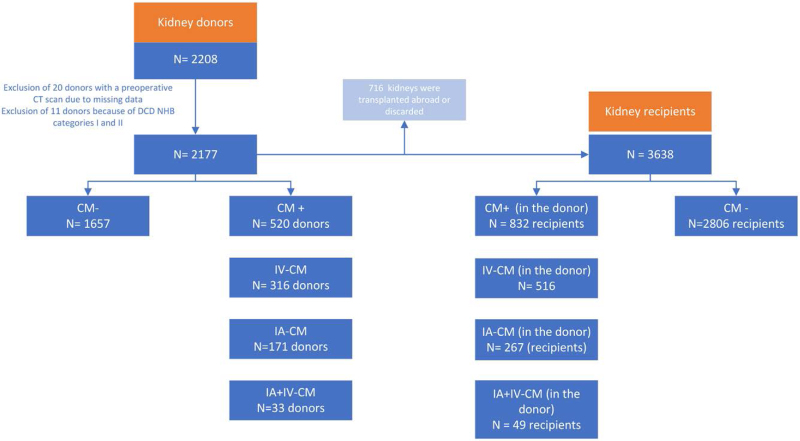
Flowchart of inclusion of donors and recipients. CM, contrast medium; IA, intra-arterial; IV, intravenous.

Between January 1, 2011, and June 30, 2021, 2208, deceased kidney donors were reported, and their organs were procured in the Netherlands, of which 31 donors had to be excluded on the basis of the previous criteria (Figure 1). This resulted in 2177 donors, of which 520 (24%) were exposed to a contrast medium. Of these, 316 donors (16%) underwent an enhanced CT scan with IV-contrast medium, 171 (8%) underwent coronary angiography with IA-contrast medium, and 33 (1.5%) underwent both. Donor characteristics, stratified by donor type (DBD or DCD), are summarized in Table 1. In the DBD donor group, the KDRI (*P* = 0.03) and cause of death (*P* < 0.01) were significantly different between the contrast medium^–^ and contrast medium^+^ groups. In the DCD donor group, age (*P* < 0.01), body mass index (BMI, *P* = 0.01), sex (*P* = 0.01), history of hypertension (*P* < 0.01), KDRI (*P* < 0.01), cause of death (*P* < 0.01), and the percentage of “kidney only” donors (*P* = 0.02) were significantly different, in favor of the contrast medium^+^ group.

**TABLE 1.** Donor characteristics

	Total donor population	Missing data	DBD CM^–^	DBD CM^+^	*P*	DCD CM^–^	DCD CM^+^	*P*
Overall	**n = 2177 (100%**)	**0%**	**n = 600 (67%**)	**n = 291 (33%**)		**n = 1057 (82%**)	**n = 229 (18%**)	
Age (y) 0–30 31–40 41–50 51–60 61–70 >71	53 ± 16 12% 7% 17% 28% 27% 9%	0%	53 ± 17 13% 8% 19% 21% 26% 14%	52 ± 14 11% 6% 18% 38% 23% 4%	0.37	53 ± 15 10% 6% 16% 31% 29% 8%	48 ± 19 23% 8% 17% 21% 25% 7%	**<0.01**
BMI (kg/m^2^)	26 ± 5	0%	25 ± 4	25 ± 4	0.13	26 ± 5	25 ± 4	**0.01**
eGFR (mL/min/1.73 m^2^)	96 ± 79	0.2%	89 ± 36	91 ± 30	0.52	97 ± 60	98 ± 63	0.87
Male	54%	0%	46%	44%	0.71	58%	68%	**0.01**
History of hypertension	26%	3%	29%	26%	0.25	26%	17%	**<0.01**
History of diabetes	6%	1%	5%	8%	0.36	6%	3%	0.29
History of smoking	56%	2%	57%	60%	0.16	57%	49%	0.08
Cause of death Circulatory death CVA*^a^* Suicide Trauma Respiratory problems*^b^* Other*^c^*	16% 49% 6% 21% 3% 5.5%	0%	9% 71% 5% 11% 1% 3%	4% 61% 2% 30% 1% 3%	**<0.01**	23% 41% 7% 16% 5% 8%	14% 17% 7% 52% 1% 9%	**<0.01**
Kidney only donor	19%	0%	3.2%	1.7%	0.21	32%	24%	**0.02**
KDRI	1.29 ± 0.30	9%	1.33 ± 0.33	1.28 ± 0.27	** 0.03**	1.29 ± 0.28	1.18 ± 0.30	**<0.01**
>1 exposures to CM	1.5%	0%		11%			0%	

Values are presented as mean ± SD or percentage.

Bold values indicate statistical siginificance of *P* values.

a CVA, including cerebral ischemia, intracerebral bleeding, subarachnoidal bleeding, subdural hematoma.

b Epiglottitis/laryngitis, status asthmaticus, not specified.

c Brain tumor, meningitis, status epilepticus, medical complication, not specified.

BMI, body mass index; CM, contrast medium; CVA, cerebrovascular accident; DBD, donation after brain death; DCD, donation after circulatory death; eGFR, estimated glomerular filtration rate; KDRI, Kidney Donor Risk Index, donor only.

### Recipient Characteristics

In total, 3638 kidney recipients were included, of which 832 (23%) received a kidney from a donor that was exposed to a contrast medium. Of these, 516 kidney transplant recipients (14%) received a kidney from a donor exposed to IV-contrast medium, 267 kidney transplant recipients (7%) received a kidney from a donor exposed to an IA-contrast medium, and 49 kidney transplant recipients (1.3%) received a kidney from a donor who was exposed to both IV- and IA-contrast media (Figure 1).

Most baseline characteristics were not significantly different between the 2 recipient groups except for age (*P* < 0.01; Table 2).

**TABLE 2.** Recipient characteristics

	Total recipient population	Missing data	CM^–^	CM^+^	*P*
Overall	n = 3638 (100%)		n = 2806 (77%)	n = 832 (23%)	
Age (y) 0–30 31–40 41–50 51–60 61–70 >71	57 ± 14 5% 8% 16% 23% 34% 13%	0%	57 ± 13 6% 7% 16% 22% 3% 14%	55 ± 13 5% 9% 19% 24% 3% 11%	**<0.01**
BMI (kg/m^2^)	27 ± 7	3%	27 ± 10	26 ± 5	0.71
Male	62%	0%	62%	62%	0.85
History of diabetes	24%	10%	23%	27%	0.13
History of cardiac disease	15%	3%	16%	14%	0.18
Kidney side of transplantation, left	50%	0%	50%	50%	0.88
Primary kidney disease Diabetes Glomerulonephritis Polycystic kidney disease Systemic (autoimmune) disease Hereditary nephritis Nephropathy caused by drugs Pyelonephritis Renal vascular disease Renal vascular disease because of hypertension Other	19% 8% 10% 10% 2% 2% 4% 5% 13% 29%		20% 8% 10% 10% 2% 2% 4% 5% 14% 28%	20% 8% 8% 9% 2% 2% 4% 3% 12% 32%	0.14

Values are presented as mean ± SD or percentage.

Bold value indicates statistical siginificance of *P* values.

BMI, body mass index; CM, contrast medium.

### Procurement Details

The number of arteries and veins per kidney, as well as the first warm ischemic time, second first warm ischemic time, and cold ischemic time, were not significantly different between the contrast medium^–^ and contrast medium^+^ groups. Cold storage, as a preservation method, was significantly higher in the contrast medium^–^ group (*P* < 0.01; Table 3).

**TABLE 3.** Procurement details

	CM^–^	CM^+^	*P*	Missing data
No. of arteries	1 artery: 76% Multiple arteries: 24%	1 artery: 76% Multiple arteries: 24%	0.75	3%
No. of veins	1 vein: 93% Multiple veins: 7%	1 vein: 92% Multiple veins: 8%	0.38	3%
Preservation method (CS vs HMP)	CS 56% HMP 44%	CS 42% HMP 58%	**<0.01**	0%
WIT, first period in DCD donors (min)	16 (13–19)	16 (13–18)	0.23	6%
WIT, second period (min)	31 (25–40)	31 (24–39)	0.88	17%
CIT (h)	13 (10–16)	12 (9–16)	0.31	21%

Values are as percentage as median (interquartile range) or percentage.

Bold value indicates statistical siginificance of *P* values.

CIT, cold ischemia time; CM, contrast medium; CS, cold storage; DCD, donation after circulatory death; HMP, hypothermic machine perfusion; WIT, warm ischemia time.

### Primary Outcomes

DGF was observed in 36% (n = 1321) and PNF in 3% (n = 122) of the recipients. Registered causes of PNF were infarction/thrombosis (n = 15, 12%), rejection (n = 9, 7%), nonviable kidney (n = 9, 7%), vascular or ureteric problems (n = 7, 6%), and graft infection (n = 1, 1%). In 5 cases (4%), the patient died shortly after transplantation. In 51% of the cases, the reason for PNF was not further specified in our data set. When comparing the distribution of immediate graft function, DGF, and PNF separately for recipients of DBD and DCD donor kidneys, no significant differences were observed between the contrast medium^–^ and contrast medium^+^ groups. (*P* = 0.15 and *P* = 0.60, respectively; Table 4). There was also no difference in the DGF duration in days. After stratification into 4 groups (contrast medium^–^, IV-contrast medium, IA-contrast medium, and IV+IA–contrast medium), no differences were observed in the incidence of DGF and PNF, both in the DBD and DCD settings (Table 5).

**TABLE 4.** Graft function in kidney recipients, stratified by donor type and CM exposure

	DBD			DCD		
Donor type	**CM^–^ (n = 986)**	**CM^+^ (n = 448)**	*P*	**CM^–^ (n = 1820)**	**CM^+^ (n = 384)**	*P*
Immediate graft function	67	68	0.15	42	45	0.60
DGF	23	20		47	42	
PNF	2	3		3	3	
Unknown/missing*^a^*	8	8		8	8	
Duration of DGF (d)	7 (2–12)	8 (3–14)	0.07	8 (4–14)	9 (5–14)	0.26

Values are presented as percentage or median (interquartile range).

a For some recipients “unknown” was reported in the database, and for some recipients nothing was filled out.

CM, contrast medium; DBD, donation after brain death; DCD, donation after circulatory death; DGF, delayed graft function; IA, intra-arterial; IV, intravenous; PNF, primary nonfunction.

**TABLE 5.** Delayed function in 8 different kidney recipient groups, depending on donor type, CM exposure, and contrast type

			DBD				DCD		
	CM^–^ (n = 986)	IV-CM (n = 145)	IA-CM (n = 254)	IA + IV CM (n = 49)	*P*	CM^–^ (n = 1820)	IV-CM (n = 371)	IA-CM (n = 13)	
Immediate graft function	67	70	66	69	0.33	42	45	62	0.72
DGF	23	19	22	12		47	43	38.5	
PNF	2	4	3	4		3	4	0	

Values are presented as percentage. No DCD donor received IA + IV CM.

CM, contrast medium; DBD, donation after brain death; DCD, donation after circulatory death; DGF, delayed graft function; IA, intra-arterial; IV, intravenous; PNF, primary nonfunction.

Univariable logistic regression showed that contrast medium^+^ kidneys had a significantly lower risk of developing DGF (OR 0.70; 95% CI, 0.59-0.83; *P* < 0.01; Table 6). In multivariable logistic regression analysis, when adjusted for potential confounding factors, contrast medium exposure did not have a significant effect on the development of DGF (OR 1.06; 95% CI, 0.86-1.36; *P* = 0.63; Table 6, models 1–3). Donor type (DCD), donor age, donor sex (male), donor cause of death, donor hypertension, recipient age, recipient BMI, recipient cardiac history, multiple arteries, preservation method, first warm ischemic time, and cold ischemic time were associated with the occurrence of DGF according to multivariate analyses (**Table S1, SDC**, http://links.lww.com/TP/C852).

**TABLE 6.** Multivariable logistic regression analysis and Cox regression analysis evaluating associations of donor, recipient, and procedural characteristics with the risk of DGF and (death-censored) graft failure in the recipient

	DGF, OR (95% CI)	*P*	Graft failure, HR (95% CI)	*P*
Univariable (CM exposure)	0.70 (0.59-0.83)	**<0.01**	1.09 (0.88-1.34)	0.42
Model 1	0.84 (0.69-1.01)	0.07	1.02 (0.81-1.23)	0.88
Model 2	1.08 (0.85-1.36)	0.52	0.97 (0.75-1.27)	0.83
Model 3	1.06 (0.83-1.36)	0.63	1.01 (0.77-1.33)	0.93

Coefficients of the full model are listed in **Table S1** (**SDC**, http://links.lww.com/TP/C852).

Model 1: CM exposure + donor age + donor BMI + donor gender + donor history of diabetes + donor history of hypertension + donor type + kidney only donor + donor cause of death.

Model 2: model 1 + first warm ischemia time 1 + second warm ischemia time + cold ischemia time + multiple arteries + multiple veins + kidney site + machine perfusion.

Model 3: model 2 + recipient age + recipient gender + recipient diabetes + recipient cardiac disease + primary disease.

Bold value indicates statistical siginificance of *P* values.

CI, confidence interval; CM, contrast medium; DGF, delayed graft function; HR, hazard ratio; OR, odds ratio.

### Secondary Outcomes

**FIGURE 2.**
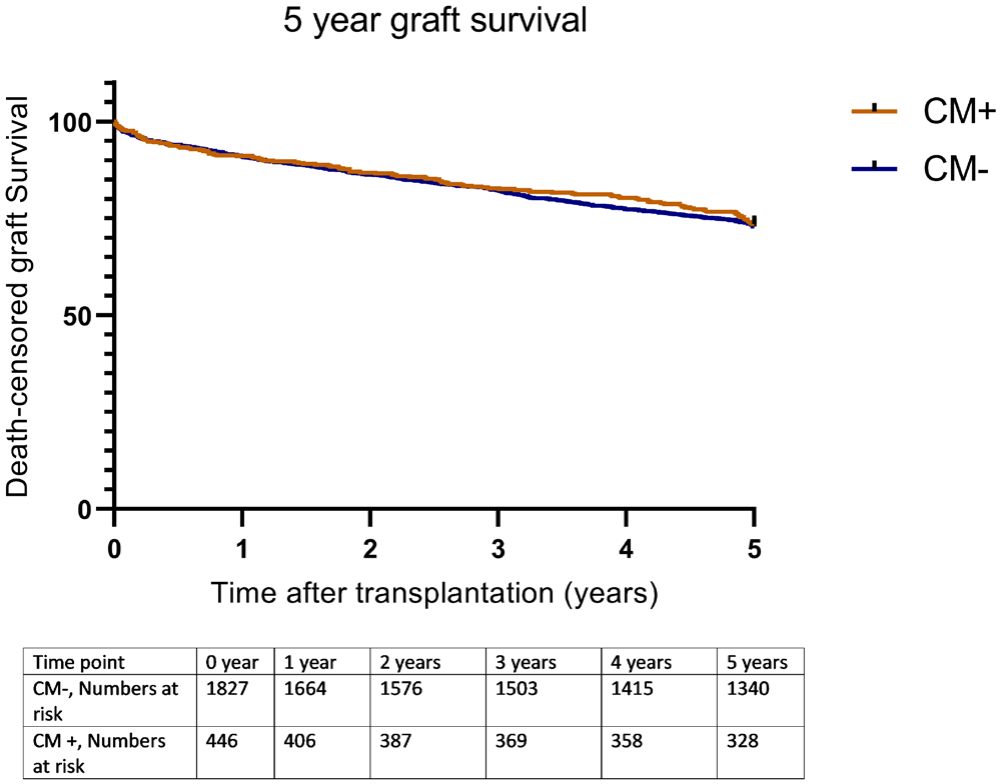
Death-censored graft survival until 5 y posttransplantation, according to contrast medium exposure of the donor (log-rank test *P* = 0.79). CM, contrast medium.

Kaplan-Meier survival analysis showed no significant differences in death-censored graft survival 5 y posttransplantation between the contrast medium^–^ and contrast medium^+^ groups (log-rank test *P* = 0.79; Figure 2).

Univariable Cox regression showed that contrast medium^+^ kidneys did not have a higher hazard rate for graft failure (HR 1.09; 95% CI, 0.88-1.34; *P* = 0.42). In the multivariable analysis, after adjustment for potential confounders, contrast medium exposure was not significantly associated with graft failure rate (HR 1.01; 95% CI, 0.77-1.33; *P* = 0.93; Table 6, models 1–3). Donor age, donor history of hypertension, DBD donation, recipient age, recipient BMI, recipient cardiac history, first warm ischemic time, and cold ischemic time were significant predictors of death-censored graft failure (**Table S1, SDC**, http://links.lww.com/TP/C852).

**FIGURE 3.**
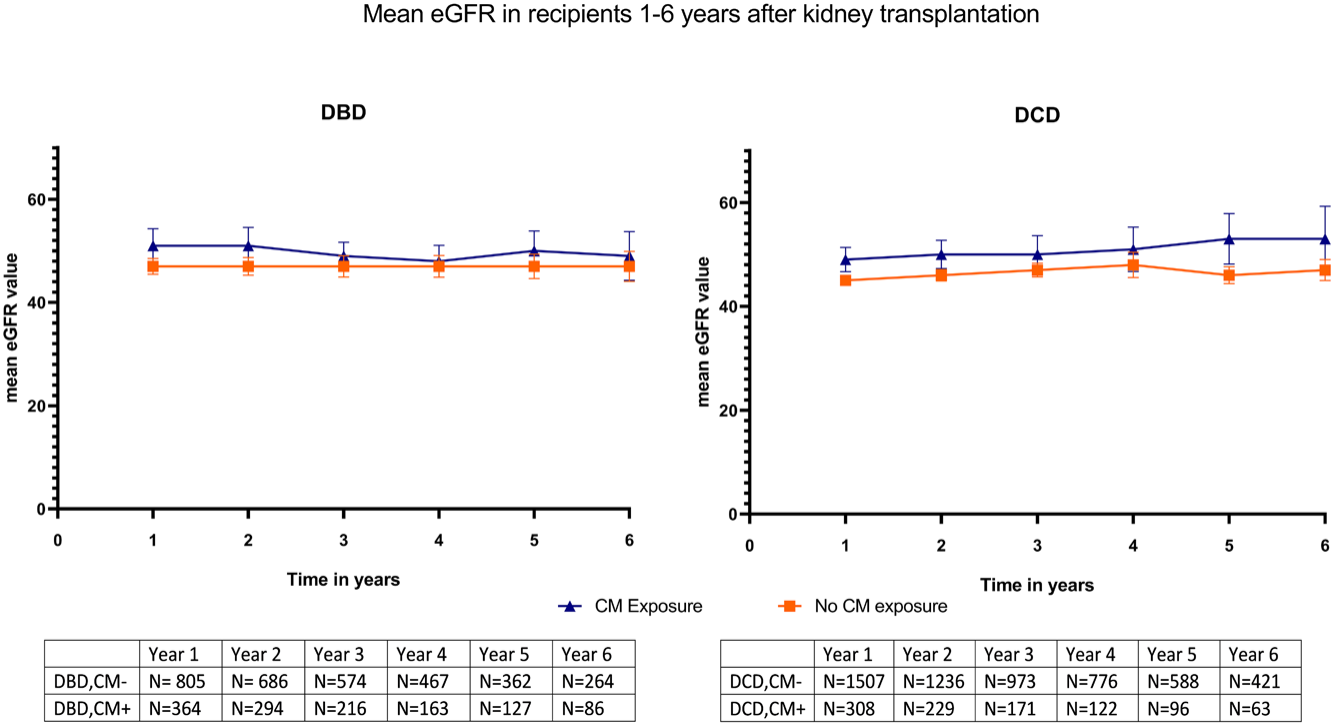
Mean eGFR (in mL/min/1.73 m^2^) in kidney recipients 1 to 6 y after transplantation. CM, contrast medium; DBD, donation after brain death; DCD, donation after circulatory death; eGFR, estimated glomerular filtration rate.

In a linear mixed model using eGFR as the dependent variable, there was no significant difference in the mean eGFR over time between the contrast medium^–^ and contrast medium^+^ group at 1 to 6 y posttransplantation (*P* = 0.78; Figure 3).

### Subanalyses of Donors With eGFR <60 mL/min/1.73 m^2^

In **Table S2** (**SDC**, http://links.lww.com/TP/C852), separate analyses for donors with an eGFR <60 mL/min/1.73 m^2^ can be found. In the data set, there were 286 donors with an eGFR <60 mL/min/1.73 m^2^, of which only 21% (n = 60) were exposed to contrast medium. Comparing baseline characteristics in donors with an eGFR <60 mL/min/1.73 m^2^, stratified by contrast medium exposure, donor type, cause of death, and KDRI significantly differed. No significant differences were observed in baseline recipient characteristics. DGF, PNF, and immediate graft function in recipients did not differ between the 2 groups.

### Subanalyses of Donors Who Received Coronary Angiography

In **Table S5** (**SDC**, http://links.lww.com/TP/C852), separate analyses for donors who underwent coronary angiography are presented. Coronary angiography was solely performed to determine suitability for heart donation, which was only possible for DBD donors until March 2022. Therefore, the comparison of donors who underwent coronary angiography with donors who did not undergo coronary angiography was limited to DBD donors, excluding “kidney only donors.” A total of 338 DBD donors in the data set donated their hearts. Of these donors, 142 (72%) underwent coronary angiography. Donors who underwent coronary angiography were significantly older (55 y ± 8 versus 51 y ± 16, *P* < 0.01), had a higher BMI (26* ±* 5 versus 25 ± 4, *P* = 0.01), and more often had a medical history of smoking (66% versus 55%, *P* = 0.02) compared with multiorgan donors who did not undergo coronary angiography (**Table S5, SDC**, http://links.lww.com/TP/C852). Most baseline characteristics were not significantly different between the 2 recipient groups except for age and history of diabetes (**Table S6, SDC**, http://links.lww.com/TP/C852). Primary outcomes (DGF, PNF, and immediate graft function) in recipients did not differ between the 2 groups (**Table S7, SDC**, http://links.lww.com/TP/C852).

### Kidneys Not Accepted for Transplantation

Between January 1, 2014, and June 30, 2021, in total 2011 potential kidney donors were reported to Eurotransplant, including nonpursued or effectuated donors. Of these, 1558 donors were included in this study, which are donors of which the kidneys were procured and transplanted in the Netherlands. The reasons for not transplanting the resulting donors were traced back in the data. Contrast medium–associated AKI has not been specifically reported as a reason for not accepting kidneys for transplantation. Donors with reported reasons indicating acute kidney injury, such as “bad kidney function,” “kidney dysfunction,” “kidney failure,” and “high creatinine” were therefore screened for contrast medium exposure. This led to the selection of 28 donors, of whom the kidneys were declined for transplantation because of the above-written reasons. Of these 28 donors, only 6 donors received contrast medium.

## DISCUSSION

This study showed that IA- and IV-contrast medium administration in organ donors is not significantly associated with a higher risk of DGF or death-censored graft failure in kidney graft recipients. We did not observe an effect of contrast medium exposure in donors on the long-term eGFR in the recipient. Separate analyses of DBD and DCD donors showed no difference in the incidence of DGF between contrast medium^–^ and contrast medium^+^. Additionally, contrast medium type (IV-contrast medium alone, IA-contrast medium alone, or IV- and IA-contrast media combined) had no effect on DGF rate. AKI, because of the administration of contrast medium, was not a frequent cause of decline of a kidney offer as far as this can be traced back in the available data. To our knowledge, this is the first study to investigate the effects of contrast medium exposure in DBD as well as DCD donors.

The indications for performing a CT scan were not registered in the Eurotransplant database. Donors with trauma as a cause of death more often underwent a CT scan. Therefore, performing a (whole body) CT scan at admission as part of trauma screening could be a frequent indication.

This was confirmed by Dutch guidelines, according to which trauma patients often receive a contrast medium-enhanced chest and/or abdominal CT scan at admission.^32^ This could also explain the mean donor age, which was significantly lower in the DCD contrast medium^+^ group than in the DCD contrast medium^–^ group (Table 1). According to the Dutch Trauma Registry, the mean age of trauma patients is 56 y (SD 30).^33^

The indication for coronary angiography in this data set was solely donor screening in the context of determining suitability for heart donation, on request of the transplant center. Therefore, it makes sense that these donors have a significantly higher mean age and more comorbidities because it is imaginable that a transplant center in these cases would want more certainty regarding the quality of the heart.

Our findings regarding contrast medium exposure in kidney donors and DGF are consistent with those in the literature. Magnus et al^19^ performed a retrospective analysis of 709 kidney donors who received IV-contrast medium and found no difference in serum creatinine levels in the donor or in DGF and graft loss in the recipients compared with 685 kidney donors who did not receive IV-contrast medium. This study group only contained DBD donors, with a median age of 35 y, which makes this study population less comparable with the current European donor populations. Lesouhaitier et al^20^ found that coronary angiography (IA-contrast medium, as part of cardiac donor evaluation), with or without other diagnostic examinations requiring IV-contrast medium administration, did not influence the DGF rate in kidney graft recipients. The study population was, in terms of age, more comparable with deceased organ donors in the Netherlands because the age of >40% of the donors was between 50 and 59 y. However, a high risk for coronary artery disease was also an inclusion criterium which most likely explains the high DGF rate (42%) in this cohort, compared with the Dutch DBD donors (DGF rate 17%).^26^ Another plausible explanation for this difference could be that only 18% of the included kidneys were procured on hypothermic machine perfusion, compared with 51% in our cohort. Hypothermic machine perfusion significantly reduces the risk of developing DGF.^34^

The major strength of our study was the relatively large and diverse cohort, including DBD and DCD donors of different age categories. Additionally, the availability of complete follow-up data on kidney recipients contributed to the reliability of our results. However, this study also has a few limitations. This was a retrospective study, which is always accompanied by loss or unavailability of data. Access to a mandatory prospective national registry has significantly minimized this risk. However, the lack of information regarding the dosage and type of contrast medium used means that it was impossible to assess a dose–effect relationship or analyze differences between various types of contrast medium. In addition to dosage, the exact timing of contrast medium administration before the donation was not available; therefore, the impact of the timing of contrast medium administration and the start of the procedure could not be investigated. Also, it would have been of interest to perform a subanalysis on the effect of contrast medium administration on donors with AKI, but because serial creatinine levels were not available, this was not possible in our study. Therefore applying the results of this study to donors with AKI requires caution. Because ethnicity is not registered in our database, we could not consider this when calculating the KDRI and eGFR values. According to the European General Data Protection Regulation, registration of ethnicity is prohibited within the European Union.^35^ Furthermore, because a contrast-enhanced CT scan is not part of the standard donor workup, the contrast medium^+^ group was relatively small compared with the contrast medium^–^ group. Nevertheless, power calculations showed that at least 752 donors were needed in each group to detect a difference of 10% in DGF, which was achieved in our cohort (contrast medium^–^ 2806 recipients versus contrast medium^+^ 832 recipients). Finally, only 13 recipients received a graft from a DCD donor exposed to IA-contrast medium, and no kidney transplant recipients received a graft from a DCD donor exposed to both IA- and IV-contrast media during the donor evaluation procedure. These small sample size made it difficult to draw conclusions. Taking all this into consideration, further research should focus on contrast dosage, contrast type, and multiple administrations of IA- and IV-contrast medium, especially in DCD donors. Also, the timing between contrast administration and the procedure should be investigated, with special attention paid to the applicability of these results to donors with AKI.

In conclusion, our results suggest that donor IA- and/or IV-contrast medium administration in DBD donors and IV-contrast medium administration in DCD donors do not affect short- and long-term outcomes after kidney transplantation and is safe for use during the organ donor evaluation process.

## ACKNOWLEDGMENTS

The authors thank all Dutch kidney transplantation centers for their contribution to the NOTR.

## Supplementary Material


