# Supplementary material for: Transplant Outcomes After Exposure of Deceased Kidney Donors to Contrast Medium
Source: Transplantation. 2023 Sep 6;108(1):252–60. doi: 10.1097/TP.0000000000004745 (PMC10718213; doi:10.1097/TP.0000000000004745)
Supplement: Supplementary file 1 [file tpa-108-252-s001.pdf]

Supplementary

Table 1: Multivariable logistic regression analysis and Cox regression analyses evaluating associations of donor, recipient, and procedural characteristics with the risk of delayed graft function and graft failure in the recipient. Results of model 3.

	DGF / **OR**	**P-Value**	**Graft failure/ HR**	
**Contrast medium exposure**	1.06 [0.83-1.36]	p=0.63	1.01 [0.77-1.33]	p=0.92
**Donor characteristics**				
Age	1.01 [1.00-1.02]	**p=0.01**	1.02 [1.01-1.03]	**p=<0.01**
BMI	1.01 [0.99-1.03]	p=0.18	0.99 [0.97-1.01]	p=0.36
Gender (male as reference)	0.80 [0.66-0.98]	**p=0.03**	0.99 [0.81-1.23]	p=0.99
History of diabetes	1.02 [0.67-1.53]	p=0.10	1.03 [0.70-1.53]	p=0.87
History of hypertension	1.42 [1.13-1.79]	**p=0.01**	1.36 [1.08-1.73]	**p=0.01**
Cause of death		**p<0.01**		p=0.61
- CVA (reference)	1		1	
- Cardiac	1.23 [0.95-1.62]		1.03 [0.76-1.40]	
- Trauma	1.08 [0.82-1.41]		1.04 [0.77-1.42]	
- Other	0.57 [0.42-0.77]		1.25 [0.90-1.73]	
DCD donor (DBD as reference)	2.16 [1.42-3.29]	**p<0.01**	0.52 [0.34-0.81]	**p=0.014**
Kidney only donor	0.97 [0.76-1.24]	p=0.82	1.00 [0.76-1.31]	p=0.99
**Recipient characteristics**				
Age	0.99 [0.98-0.99]	**p=0.01**	0.98 [0.98-0.99]	**p<0.01**
BMI	1.03 [1.01-1.05]	**p=0.01**	1.01 [1.00-1.02]	**p=0.02**
Gender	0.83 [0.68-1.00]	p=0.06	1.11 [0.90-1.38]	p=0.33
History of diabetes	1.29 [0.97-1.72]	p=0.19	1.31 [0.96-1.79]	p=0.09
History of cardiac disease	1.72 [1.32-2.24]	**p<0.01**	1.30 [0.98-1.72]	**p<0.01**
Primary disease		p=0.33		p=0.10
- Polycystic disease (reference)	1		1	
- Diabetes Mellitus	0.86 [0.55-1.35]		1.23 [0.71-2.13]	
- Glomerulonephritis	1.04 [0.66-1.62]		1.41 [0.83-2.41]	
- Systemic (auto-immune) disease	0.93 [0.61-1.43]		1.27 [0.74-2.17]	
- Renal vascular disease	1.06 [0.73-1.54]		1.83 [1.15-2.89]	
- Other	0.80 [0.57-1.12]		1.48 [0.95-2.30]	
**Transplant characteristics**				
Cold ischemic period	1.04 [1.02-1.05]	**p<0.01**	1.02 [1.01-1.04]	**p=0.01**
First warm ischemic time	1.04 [1.02-1.07]	**p<0.01**	**1.04 [1.02-1.06]**	**p=0.01**
Second warm Ischemic time	1.00 [0.99-1.01]	p=0.31	**1.00 [0.99-1.01]**	p=0.85
Multiple arteries	1.41 [1.13-1.76]	**p=0.02**	1.23 [0.97-1.55]	p=0.09
Multiple veins	1.03 [0.73-1.47]	p=0.86	0.91 [0.61-1.34]	P=0.63
Kidney site (left as reference)	0.95 [0.79-1.15]	p=0.59	0.92 [0.75-1.13]	p=0.45
Preservation method (CS as reference)	0.59 [0.48-0.72]	**P<0.01**	0.90 [0.71-1.13]	P=0.35

Table 2: Donor characteristics of donors with an eGFR <60mL/min/1.73m2.

	**Donors with eGFR <60, CM –**	**Donors with eGFR<60, CM +**	
**Overall**	N= 226 (79%)	N=60 (21%)	
**Donor type, DBD**	N= 95 (42%)	N=34 (N=57%)	**p=0.04**
**Age (in years)**<br>-	54 $\pm$ 13	51 $\pm$ 15	p=0.11
**BMI (kg/m^2)**	26 $\pm$ 5	26 $\pm$ 4	p=0.88
**eGFR (mL/min/1.73m^2)**	51 $\pm$ 8	50 $\pm$ 8	p=0.61
**Gender, Male**	47%	48%	p=0.84
**History of hypertension**	27%	16%	p=0.08
**History of diabetes**	1%	0%	p=0.48
**History of smoking**	53%	62%	p=0.23
**Cause of death**<br><br>Cardiac death<br>CVA *<br>Suicide<br>Trauma<br>Respiratory problems**<br>Other***	 <br>38%<br>40%<br>11%<br>7%<br>3%<br>2%	 <br>23%<br>33%<br>12%<br>25%<br>2%<br>5%	**p=0.01**
**Kidney only donor**	20%	23%	p=0.56
**KDRI**	1.41	1.30	**p=0.02**

Values are presented as mean ($\pm$SD) or percentage
**Cerebral ischemia, intracerebral bleeding, subarachnoidal bleeding, subdural hematoma*
***Epiglottitis/laryngitis, status asthmaticus, not specified*
****Brain tumor, meningitis, status epilepticus, medical complication, not specified*
CM= Contrast medium exposure. KDRI = kidney donor risk index, donor-only

Table 3: Recipient characteristics of recipients who received a graft from a donor with an eGFR<60

	Total recipient population	CM -	CM +	
**Overall**	N= 468	378	90	
**Age (in years)**	58 ± 13	58± 13	59 ± 13	p=0.47
**BMI (kg/m^2)**	27 ±5	27 ± 5	27 ±5	P=0.81
**Gender, Male**	63%	64%	60%	P=0.54
**History of diabetes**	25%	26%	22%	p=0.36
**History of cardiac disease**	16%	17%	9%	p=0.14
**Kidney side of transplantation, Left**	50%	50%	48%	P=0.64
**Primary kidney disease**				p=0.23
Diabetes Mellitus	18%	20%	11%	
Glomerulonephritis	7%	7%	7%	
Systemic (auto-immune) disease	11%	11%	8%	
Renal vascular disease	16%	15%	17%	
Polycystic kidney disease	11%	9%	16%	
Other	38%	37%	42%	

CM= Contrast medium exposure. Values are presented as percentage

Table 4: Graft function in kidney recipients who received a graft from a donor with an eGFR<60, stratified by contrast medium exposure of the donor

	**CM-**	**CM+**	
**Immediate graft function**	54%	57%	p=0.59
**Delayed graft function**	42%	37%	
**Primary Non function**	2%	5%	
**Unknown/missing**	2%	1%	

CM= Contrast medium exposure. Values are presented as percentage

Table 5: Donor characteristics, only including multi organ donors, stratified by if the donor underwent coronary angiography (intra-arterial contrast medium exposure) or not

	**Total multi organ donor population**	**Coronary angiography-**	**Coronary angiography +**	
**Overall**	N=868	N=670	198	
**Age (in years)**	52 ± 16	51 ± 16	55 ± 8	**p<0.01**
**BMI (kg/m²)**	25 ± 4	25±4	26 ±5	**p=0.01**
**eGFR (mL/min/1.73m²)**	90 ± 34	89 ± 35	93 ± 29	p=0.15
**Gender, Male**	45%	47%	40%	p=0.12
**History of hypertension**	29%	28%	32%	p=0.33
**History of diabetes**	2%	1.4%	2.2%	p=0.46
**History of smoking**	57%	55%	66%	**p=0.02**
**Cause of death**				**p<0.01**
Cardiac death	7%	8%	2%	
CVA *	68%	66%	77%	
Suicide	3%	5%	2%	
Trauma	18%	18%	17%	
Respiratory problems**	1%	1%	1%	
Other***	3%	4%	2%	
**KDRI**	1.31 ± 0.32	1.30 ± 0.34	1.33 ± 0.20	**p=0.01**
**Heart donor**	N= 338 (40%)	N= 196 (29%)	N=142 (72%)	**p<0.01**

Values are presented as mean (±SD) or percentage

Table 6: Recipient characteristics, only including recipients who received a graft from a multi organ donor, stratified by if the donor underwent coronary angiography (intra-arterial contrast medium exposure) or not

	**Coronary angiography-**	**Coronary angiography +**	
**Overall**	N=1089 (78%)	N=303 (22%)	
**Age (in years)**	56 ± 15	54 ± 13	**p=0.01**
**BMI (kg/m²)**	26 ± 5	26 ± 5	p=0.68
**Gender, Male**	62%	63%	p=0.80
**History of diabetes**	23%	27%	**p=0.03**
**History of cardiac disease**	14%	14%	p=0.96
**Kidney side of transplantation, Left**	52%	51%	p=0.61
**Primary kidney disease**			p=0.05
Diabetes Mellitus	23%	21%	
Glomerulonephritis	7%	9%	
Systemic (auto-immune) disease	9%	7%	
Renal vascular disease	18%	15%	
Polycystic kidney disease	10%	7%	
Other	33%	42%	

Values are presented as mean (±SD) or percentage

Table 7: Graft function in kidney recipients, who received a graft from a multi organ donor, stratified by if the donor underwent coronary angiography (intra-arterial contrast medium exposure) or not

	Coronary angiography-	Coronary angiography +	
**Immediate graft function**	73%	72%	p=0.60
**Delayed graft function**	23%	22%	
**Primary Non function**	3%	3%	
**Unknown/missing**	1.5%	2.5%	

Values are presented as percentage.
